# Direct and indirect costs of tuberculosis among immigrant patients in the Netherlands

**DOI:** 10.1186/1471-2458-9-283

**Published:** 2009-08-05

**Authors:** Sandra V Kik, Sandra PJ Olthof, Jonie TN de Vries, Dick Menzies, Naomi Kincler, Joke van Loenhout-Rooyakkers, Conny Burdo, Suzanne Verver

**Affiliations:** 1 Research Unit, KNCV Tuberculosis Foundation, The Hague, the Netherlands; 2 Center for Infection and Immunity Amsterdam, Academic Medical Center Amsterdam, the Netherlands; 3 Respiratory Division, McGill University, Montreal, Canada; 4 Department of Tuberculosis Control, Municipal Health Service Regio Nijmegen, the Netherlands; 5 Department of Tuberculosis Control, Municipal Public Health Service Rotterdam-Rijnmond, Rotterdam, the Netherlands

## Abstract

**Background:**

In low tuberculosis (TB) incidence countries TB affects mostly immigrants in the productive age group. Little empirical information is available about direct and indirect TB-related costs that patients face in these high-income countries. We assessed the direct and indirect costs of immigrants with TB in the Netherlands.

**Methods:**

A cross-sectional survey at 14 municipal health services and 2 specialized TB hospitals was conducted. Interviews were administered to first or second generation immigrants, 18 years or older, with pulmonary or extrapulmonary TB, who were on treatment for 1–6 months. Out of pocket expenditures and time loss, related to TB, was assessed for different phases of the current TB illness.

**Results:**

In total 60 patients were interviewed. Average direct costs spent by households with a TB patient amounted €353. Most costs were spent when being hospitalized. Time loss (mean 81 days) was mainly due to hospitalization (19 days) and additional work days lost (60 days), and corresponded with a cost estimation of €2603.

**Conclusion:**

Even in a country with a good health insurance system that covers medication and consultation costs, patients do have substantial extra expenditures. Furthermore, our patients lost on average 2.7 months of productive days. TB patients are economically vulnerable.

## Background

Tuberculosis (TB) affects people within the most productive age group and the resultant economic costs for society are high [1,2]. Previous studies on the economic aspects of TB control have focused on health system costs for interventions like screening, or on evaluation of these interventions [3-10]. There is little information about the TB related costs incurred by TB patients and their households throughout the entire illness, including the pre-diagnostic phase. The only studies assessing patient and household costs have been conducted in high TB prevalence and low-income countries [6,11-16], but are lacking for low-prevalence and high-income countries. Health policy makers need this information to assess the overall economic impact of TB. Even though in most countries diagnostic and treatment services for TB control are offered free of charge, TB patients do have out of pocket expenditures [11,12].

As in many other low-incidence countries, in the Netherlands, immigrants and refugees contribute to a high TB case load, compared to native inhabitants [3,17,18]. In 2007, among the 960 registered TB patients in the Netherlands 592 (61%) were foreign-born. Enrolment in a private health insurance is compulsory for all inhabitants in the Netherlands. Beside the mandatory basic health insurance (approximately €95 per month), people can additionally choose for a more extended insurance with a higher coverage. Moreover, individuals with a yearly gross income below €26071 qualify for a health care allowance of maximum €36 per month. In 2007, the basic health insurance covered all costs of TB medication, most diagnostic tests, doctors' consultation and hospitalisation, but not costs related to travel and food supplements etc. Since immigrants are often faced with lower annual incomes than the non-migrant population, TB related expenditures not covered by the health insurance might be disproportionately higher for immigrants than for non-migrant patients.

In this study we describe the direct and indirect costs of TB among immigrants with TB disease in the Netherlands.

## Methods

### Study design and population

Between April 2007 and October 2007 we conducted a cross-sectional survey at the 14 municipal health services (MHSs) and the two specialized TB hospitals that volunteered to participate in the Netherlands. They were selected based on the largest number of patients, being usually from larger cities. This study was part of a multi-centre international study on cost-effectiveness of worldwide interventions, coordinated by McGill University in Montreal, Canada. In this paper we present the data collected in the Netherlands.

We included tuberculosis patients who were a first or second generation immigrants, who were on anti-TB treatment and aged 18 years or older. First generation immigrants were those individuals born in a foreign country and who were living in the Netherlands at the time of diagnosis of active TB. Second generation immigrants were those individuals who were born in the Netherlands, but who had at least one parent that was born abroad.

### Data collection

Patients who fulfilled the inclusion criteria were invited to participate in the study by the nurse from the MHS or specialized TB hospital. One of two trained interviewers explained the study, obtained informed consent and performed an in-depth face-to-face interview. A structured questionnaire was used, developed by McGill University (Montreal, Canada), that had been successfully used in previous studies [6,10,11], and was adapted to the Dutch setting. Patients who were unable to speak Dutch or English were helped by family members or friends who could speak one of these two languages. If necessary a translator assisted by telephone. Interviews were done in a private room, without the presence of any medical staff in order to guarantee the patients' privacy.

Medical information from all TB patients in the Netherlands is registered in the Netherlands Tuberculosis Register (NTR). The NTR-committee and all participating MHSs gave permission to extract anonymous medical data from this register for the study population. To verify if our study population was comparable to all TB patients we compared our study population with all TB patients (who fulfilled the study inclusion criteria) who were diagnosed in 2005. Since complete national data were at the time of the study only available for 2005 we assumed that TB cases notified in the NTR in 2005 were comparable to those of 2007. No ethical approval was needed for this study from the Central Committee on Research Involving Human Subjects (CCMO) as our questionnaire did not involve any intrusive questions.

### Costs assessed

Information about costs was ascertained for different periods starting from the moment of onset of symptoms up to the completion of treatment. Direct costs and time loss during the different periods were asked per visit. Since patients were still under treatment at the moment of the interview, we assumed that the length of their treatment would be comparable with the average treatment length of immigrant patients in 2005; which was 6.4 months for the whole treatment. Patients who received directly observed therapy (DOT) received their medication 3 to 5 times per week at the MHS. The duration of DOT was on average 5.0 months in 2005 according to the Dutch surveillance data. All costs were extrapolated for the entire treatment period.

### Direct costs

Direct patient costs included all out of pocket expenditures of patients that were attributable to their illness [19]. Direct costs included the costs of transportation to health facilities, costs of extra food (bought during waiting time at the health facility), extra expenditures related to hospitalization (which were not covered by the health insurance) or over the counter medication (such as vitamin B6). These costs were assessed both for the patient as well as for their household members when they had extra expenditures due to the illness of the patient.

### Indirect costs

Indirect patient costs refer to the costs associated with time lost by the patient. This loss of time included visits to the health facilities and hospitalization as well as other time lost due to the inability to work as a result of the illness [19]. Time loss was only collected for the patients themselves. In order to quantify the magnitude of time loss, the number of days lost was multiplied by the estimated income of the patients. Because of the sensitivity of the question, we did not ask the participants their exact income, but asked them to indicate which of 4 categories their income corresponded to. To quantify the indirect costs, the average time loss, in days, was multiplied by the estimated average income of the study population.

### Data and statistical analysis

Questionnaires were double entered by two different persons into a standardized database. Data were analysed with SPSS 14.0. Costs and time loss were compared between patients with pulmonary tuberculosis (PTB) and patients with extrapulmonary tuberculosis (ETB). Patients with both PTB and ETB were classified as PTB. Odds ratio's (OR) for being included in our study compared to all immigrant patient (regardless of their inclusion on our study) were determined by logistic regression analysis, to determine the representativeness of the patients included in our study for all immigrant patients in 2005. P-values were calculated by chi-square test, independent t-test or non-parametric tests as appropriate. While data on costs and time loss was not normally distributed, we reported mean values to allow comparison with other published cost estimates. In our tables also median values are given.

## Results

### Patient characteristics

During the study period, 60 patients fulfilled the inclusion criteria and consented to participate. In general, our study population included more males (63%) than females (37%). The majority of the patients included in the study were 25 to 44 years old and most patients were first generation immigrants from Africa or Asia (Table 1). Compared to all immigrant patients diagnosed in the Netherlands in 2005, our study population was slightly younger and included significantly fewer persons with impaired immunity (i.e. patients with HIV, diabetes, malignancy, an organ transplantation, renal insufficiency or other causes of immune suppression or who used TNF-α inhibitors). None of the four interviewed patients with an impaired immunity was known to be HIV positive.

**Table 1:** Comparison between the interviewed immigrants in the study and all immigrants with tuberculosis diagnosed in 2005

	Immigrant TB patients in 2005, N (%)*	Study population, N (%)*	*P-value Chi-square*
**Total**	762 (100)	60 (100)	
**Sex**			0.49
Male	448 (59)	38 (63)	
Female	314 (41)	22 (37)	
**Age (yr)**			0.08
18–24	150 (20)	18 (30)	
25–44	386 (51)	33 (55)	
45–64	154 (20)	8 (13)	
≥ 65	71 (9)	1 (2)	
Unknown	1 (0)	0 (0)	
**Generation**			0.43
First generation immigrant	718 (94)	58 (97)	
Second generation immigrant	44 (6)	2 (3)	
**Region of origin**			0.44
Europe	92 (12)	3 (5)	
America (Central and South)	72 (9)	6 (10)	
America (North)	1 (0)	0 (0)	
Asia	249 (33)	18 (30)	
Africa	338 (44)	33 (55)	
Unknown	10 (1)	0 (0)	
**Localisation of TB**			0.69
Pulmonary	357 (47)	31 (52)	
Extrapulmonary	305 (40)	23 (38)	
Pulmonary and extrapulmonary	100 (13)	6 (10)	
**Culture result**			0.83
Positive	531 (70)	44 (73)	
Negative	91 (12)	6 (10)	
Not done/unknown	140 (18)	10 (17)	
**Previously diagnosed with TB**			0.13
No	611 (80)	54 (90)	
Yes	46 (6)	3 (5)	
Unknown	105 (14)	3 (5)	
**Treatment regime**			< 0.01
Standard (4HRZ(E)/2HR(E))	542 (71)	43 (72)	
Other	220 (29)	9 (15)	
Unknown	0 (0)	8 (13)	
**Impaired immunity**			< 0.01
No	282 (37)	40 (67)	
Yes	140 (18)	4 (7)	
Missing	340 (45)	16 (27)	
**Hospitalization (during TB treatment for at least 7 days)**			0.16
No	438 (58)	29 (48)	
Yes	232 (30)	19 (32) ‡	
**Unknown**	92 (12)	12 (20)	

Definition of abbreviations: H = isoniazid; R = rifampin; Z = pyrazinamid; E = ethambutol;

* Column percentages are given

‡ This number differs from that in Table 2 because a different definition for hospitalization is used in the NTR-database.

Out of 60 patients, 31 (52%) had PTB, 23 had (38%) ETB and 6 (10%) suffered from both PTB and ETB. We did not observe any significant differences between characteristics of the interviewed patients with PTB and ETB, but ETB patients tended to be older than PTB patients (Table 2). Out of 60 participants, 34 (57%) were unemployed at the time of the interview. Nine (26%) of the 34 unemployed patients were asylum seekers, who are not allowed to have a paid job during their entrance procedure. Eight other patients (13%) stated that their unemployment was due to their TB illness. A lot of the patients (48%) were living alone in a household. Forty three percent of the immigrants with TB reported that their net household income before diagnosis was less than €1000 per month. Post-diagnosis this percentage increased slightly to 50%. Six patients reported a reduction of their income, while 3 reported an increase since their diagnosis. Except for 2 patients, 58 (97%) had a health insurance.

**Table 2:** Socio-economic characteristics of immigrants with pulmonary tuberculosis compared to immigrants with extrapulmonary tuberculosis

	All TB cases, N (%)*	Pulmonary TB cases, N (%)*‡	Extrapulmonary TB cases, N (%)*	*p-value†*
**Total**	60	37	23	
**Sex**				
Male	38 (63)	24 (65)	14 (61)	0.76
Female	22 (37)	13 (35)	9 (39)	
**Age (yr)**				
18–24	18 (30)	13 (34)	5 (23)	0.06
25–44	33 (55)	23 (61)	10 (46)	
45–64	8 (13)	2 (5)	6 (27)	
≥ 65	1 (2)	0 (0)	1 (5)	
**Region of origin**				
Europe	3 (5)	3 (8)	0 (0)	0.25
America (Central and South)	6 (10)	2 (5)	4 (18)	
Asia	18 (30)	12 (32)	6 (27)	
Africa	33 (55)	21 (55)	12 (54)	
**Immigration status**				
Landed immigrant	33 (55)	15 (41)	18 (79)	0.12
Immigrant applicant (asylum seeker)	12 (20)	10 (27)	2 (9)	
Illegal immigrant	4 (7)	3 (8)	1 (4)	
Accepted refugee	3 (5)	2 (5)	1 (4)	
Second generation immigrant	2 (3)	2 (5)	0	
Student	6 (10)	5 (14)	1 (4)	
**Level of education**				
No education	6 (10)	5 (14)	1 (5)	0.53
Primary school	11 (18)	6 (15)	5 (22)	
Some high school (not completed)	10 (17)	5 (14)	5 (22)	
High school	18 (30)	10 (27)	8 (35)	
Above high school	15 (25)	11 (30)	4 (17)	
**Employment status**				
Employed or student	26 (43)	16 (43)	10 (43)	0.99
Unemployed	34 (57)	21 (57)	13 (57)	
**Unemployment due to TB (n = 34)**				
Yes	8 (24)	5 (24)	3 (23)	0.96
No	26 (76)	16 (76)	10 (77)	
**Pre-diagnostic period;** **Monthly household income (after tax rebate)**				
< €100	4 (7)	4 (11)	0	0.27
€100 – €499	10 (17)	6 (16)	4 (17)	
€500 – €999	12 (20)	5 (14)	7 (30)	
≥ €1000	33 (55)	21 (57)	12 (52)	
Unknown	1 (2)	1 (3)	0 (0)	
**Post-diagnostic period;** **Monthly household income (after tax rebate)**				
< €100	2 (3)	2 (5)	0	0.82
€100 – €499	14 (23)	9 (24)	5 (22)	
€500 – €999	14 (23)	8 (22)	6 (26)	
≥ €1000	28 (47)	17 (46)	11 (48)	
Unknown	2 (3)	1 (3)	1 (4)	
**Difference in income after diagnosis of TB**				
Yes, income increased	3 (5)	2 (5)	1 (4)	0.98
Yes, income decreased	6 (10)	4 (11)	2 (9)	
No, no change in income	49 (82)	30 (81)	19 (83)	
Unknown	2 (3)	1 (3)	1 (4)	

* Column percentages are given

† P-values are calculated with the Chi-square test

‡ 6 patients with PTB and ETB, were classified as PTB.

### Direct patient costs

**Figure 1:**
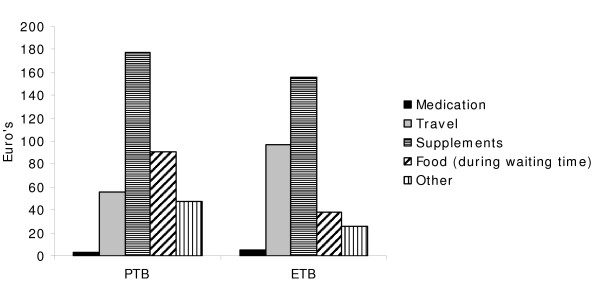
**Type of direct costs incurred by immigrants with pulmonary or extrapulmonary tuberculosis (mean costs in euro's) during the whole TB illness period**. Definition of abbreviations: PTB = pulmonary tuberculosis; ETB = extrapulmonary tuberculosis.

Direct costs of immigrants during the entire TB illness averaged €353 (median €190) (Table 3). Almost all patients (58/60) had some direct costs during their illness. Expenditures of patients varied widely and total direct costs of patients ranged from €0 to €3961. Most patients had out of pocket expenditures during the follow-up visits and the diagnostic period. Costs during the pre-diagnostic period were slightly higher for patients with ETB (mean €10, sd 18.8) compared to patients with PTB (mean €3, sd 7.4, t-test p-value = 0.04). No differences in costs during the other periods were observed for patients with different types of tuberculosis. Patient delay, defined as the period between first symptoms and first contact with a health care provider, did not differ between PTB patients (mean 2.5 months, sd 6.9) and ETB patients (mean 3.0 months, sd 4.7, p = 0.79). Most costs were incurred if patients were hospitalized. While 28 (47%) patients had been hospitalized before, during or after their TB diagnosis; mean costs during hospitalization were €105 on average. During hospitalization most costs were spent on food and other items such as the use of a TV or telephone. Additional costs consisted mainly of additional vitamins, energy drinks and food supplements. One patient reported to have extra high rent costs due to TB, which explains the maximum of €2400. Although in general costs for laboratory tests, consultation and most medication are covered by the health insurance, some patients reported that they had incurred costs on these items. In general, direct costs were spent on additional food supplements, travel to visits the health facilities and food that was bought during waiting times (Figure 1).

**Table 3:** Mean direct costs and time loss of immigrants with tuberculosis during different periods of their illness (n = 60)

	**Direct costs (euro's)**			
	Patients reporting costs during this period		All patients (n = 60)	
	N (%)*	Mean costs (min-max)†	Mean costs †	Median costs (IQR)

Pre-diagnostic period	23 (38)	€15 (€1–€72)	€6	0 (0–4)
Diagnostic period	36 (60)	€27 (€1–€190)	€16	5 (0–16)
Follow-up visits	49 (82)	€49 (€5–€195)	€40	27 (10–60)
DOT visits	13 (22)	€72 (€3–€417)	€16	0 (0–0)
Hospitalization	21 (35)	€301 (€2–€3960)	€105	0 (0–51)
Additional costs	39 (65)	€263 (€4–€2400)	€171	64 (0–251)
**TOTAL**	**58 (97)**	**€365 (€3–€3961)**	**€353**	**190 (74–399)**

	**Time loss (days)**			

	Patients reporting time loss during this period		All patients (n = 60)	
	N (%)*	Mean time loss, days (min-max)	Mean time loss	Median time loss (IQR)

Pre-diagnostic period	41 (68)	0.2 (0.0–1.8)	0.1	0.1 (0–0.2)
Diagnostic period	42 (70)	0.3 (0.0–1.2)	0.2	0.2 (0–0.4)
Follow-up visits	60 (100)	0.6 (0.18–2.0)	0.6	0.4 (0.3–0.8)
DOT visits	31 (52)	1.3 (0.2–6.6)	0.7	0.1 (0–0.7)
Hospitalization	28 (47)	39.9 (1.0–180.0)	18.6	0 (0–21.0)
Other workdays lost	39 (65)	93.0 (21.0–600.0)	60.5	31.0 (0–90.0)
**TOTAL**	**60 (100)**	**80.7 (0.5–637.2)**	**80.7**	**59.7 (4.6–121.5)**

Definition of abbreviations: IQR = inter-quarter range; DOT = directly observed therapy

* Column percentages are given

† Costs are rounded to the nearest integer.

### Indirect costs

On average patients lost 81 days of normal productivity due to their TB illness (median 60 days) (Table 3). All patients had time loss (ranging from 0.5–637 days), mainly due to travel and consultation time at different health care facilities. ETB patients had on average significantly more time loss during their pre-diagnostic period than PTB patients (mean = 0.24 vs. 0.07 days respectively, t-test p-value = 0.01). There was no difference between the time loss during other phases of the disease between ETB and PTB patients. The inability to work accounted for most time loss per patient and was on average 60.5 days. Hospitalizations led to an additional 18.6 days of time loss and visits to health facilities accounted for 1.6 days.

The Statistics Netherlands (CBS) estimated the average national disposable yearly income per household (after tax rebate and reduction of social insurance and health insurance) at €29,400 in the Netherlands in 2005 [20]. Since half of the patients in our study reported their income to be less than €1000 per month, we decided that a yearly household income of €12,000 (12*€1000) would be a more appropriate estimate of the average income of the participants in our study. To quantify the indirect costs, the average time loss, in days, was multiplied by €32.25 (€1000/31; making no difference between time loss during working days and weekend days). Consequently, the mean indirect costs corresponded with €2603 per patient.

## Discussion

This study demonstrates the economic burden that immigrant TB patients in the Netherlands face during their TB illness. We show that, even in a high income country where most medical costs are covered by the mandatory health insurance, immigrants with tuberculosis do have extra out of pocket expenditures. Furthermore, immigrant patients lost 81 productive days due to their illness. Costs and time loss during the pre-diagnostic period were slightly higher for patients with ETB compared to those with PTB, which is possibly due to the added investigations needed to diagnose extrapulmonary tuberculosis and exclude other pathology. Our study confirms that TB patients are from vulnerable groups, having a high unemployment rate both before their disease and even higher during disease, and a lower average income than the general population.

Out of pocket expenditures were comparable with 3% (€353/€12000) of the average annual income of the patients. These costs may be a considerable part of their disposable income after the payment of all monthly fixed costs such as rent, marginal costs (such as electricity and water) and insurances. Moreover, these costs are all faced during the time of TB disease and thus spend in a period of around 6 months. A quarter of the interviewed TB patients were asylum seekers, who often have even less money to spend. Some authors have concluded that a cost burden greater than 10% will be catastrophic for the household [15,21]. It is debatable if this percentage can be applied to both low and high income countries. The amount of extra costs spend will depend on the available income. For example, quite a number of patients reported to spend extra money on food supplements. It can be questioned whether patients with little income will (or need to) spend costs on these additional items, when they can't pay them. Furthermore, coping strategies may have resulted in differences in expenditures between patients, but we did not assess how patients handled these extra expenditures.

Opposed to our study, earlier studies on direct and indirect costs due to TB were done in low-income countries. While the total direct and indirect costs of our study population accounted for approximately 25% of their annual income (€353+€2956/€12000*100%), the total costs accounted for a considerable higher percentage in these low-income countries [6,11,14,15]. For example, a survey in Haiti estimated out of pocket expenses and lost income due to TB illness to be 76% of the average per capita income of Haitians, while a study from India showed that TB related costs accounted for 40% of the household income during TB illness [6,14]. The differences between these studies and ours may be explained by different social background, insurance system and a higher income per inhabitant in our high-income country.

Although time loss had not affected the income of all interviewed tuberculosis patients we did express the loss of time in costs. The height of the indirect costs may therefore be an overestimation of the costs faced by the individual patient, since these costs are mostly carried by the society. In the Netherlands, the social security system provides a benefit to all inhabitants who are jobless. Furthermore, individuals who have a job and become ill, will mostly receive their regular income. However, 57% of the interviewed immigrant patients was jobless and a quarter of them attributed their unemployment to their tuberculosis illness. An additional ten percent of the employed patients reported that their household income decreased due to their TB illness. The unemployment rate among TB patients even before their disease is higher than the national unemployment rate among non-western immigrants living in the Netherlands (16%). This in turn is much higher than that among the autochthonous Dutch population (4%). Consequently, their estimated income levels differ. Although we studied the TB related costs only among immigrant patients, it can be expected that these costs will not be much different for other TB patients, since most expenditures were spent on travel and food related items. However, when expressed as a percentage of the income, these TB related expenditures will be a smaller proportion of the income among the autochthonous TB patients.

Our study had some limitations. First of all our study population, a convenience sample of TB patients of the participating centres, was not completely representative for all immigrant TB patients. We included younger patients and fewer patients with an impaired immunity. Both factors may have led to an underestimation of the cost estimates, since older patients and those who are immunocompromised may have atypical presentations of TB, delayed diagnosis and more complex treatment needing more medical attention during follow-up. Secondly, many of the interviewed immigrants were unable to speak Dutch or English. This made it sometimes difficult to obtain reliable information, but on the basis of a face-to-face interview and with the aid of a translator the interviewers were still able to obtain detailed information about the patient costs. Face-to-face interviews are a good method to obtain detailed information from the past and diminish recall bias [22].

Lastly, we did not ask for the exact income details. In other studies the Gross National Product (GNP) and data from surveys by National Statistics Office were used [6,12]. The average income we used in this study may be underestimated since we did not know the maximum income in the highest income category. If so, this will have resulted in an underestimation of the indirect costs, and have led to an overestimation of the relative direct costs expressed as a percentage of the average income.

## Conclusion

In conclusion, this study showed that immigrants with TB are economically burdened in the Netherlands. Even in a country with a good health insurance system that covers medication and consultation costs, patients do have substantial extra expenditures. TB patients are economically vulnerable.

## Competing interests

The authors declare that they have no competing interests.

## Authors' contributions

SK participated in study design, performed statistical analysis, and wrote the draft manuscript. SO participated in the interviews of the patients, performed data entry and analysis, and wrote the report that was the basis of this manuscript. JV participated in the interviews of the patients, performed data entry and analysis, and wrote the report that was the basis of this manuscript. DM conceived the study and gave critical comments on the draft manuscript. NK conceived the study, trained the interviewers and coordinated the data collection, and gave critical comments on the draft manuscript. JLR participated in the data collection, and gave critical comments on the draft manuscript. CB participated in the data collection and gave critical comments on the draft manuscript. SV participated in the study design, coordinated the study and gave critical comments on the draft manuscript. All authors read and approved the final manuscript.

## Pre-publication history

The pre-publication history for this paper can be accessed here:


